# Effect of Different Fermentation Condition on Estimated Glycemic Index, In Vitro Starch Digestibility, and Textural and Sensory Properties of Sourdough Bread

**DOI:** 10.3390/foods10030514

**Published:** 2021-03-01

**Authors:** Hilal Demirkesen-Bicak, Muhammet Arici, Mustafa Yaman, Salih Karasu, Osman Sagdic

**Affiliations:** 1 Department of Nutrition and Dietetics, Faculty of Health Sciences, Istanbul Yeni Yuzyıl University, Istanbul 34010, Turkey; 2 Department of Food Engineering, Faculty of Chemical and Metallurgical Engineering, Yildiz Technical University, Istanbul 34210, Turkey; muarici@yildiz.edu.tr (M.A.); skarasu@yildiz.edu.tr (S.K.); sagdic@gmail.com (O.S.); 3 Department of Nutrition and Dietetics, Faculty of Health Sciences, Istanbul Sabahattin Zaim University, Istanbul 34303, Turkey; mustafa.yaman@izu.edu.tr

**Keywords:** sourdough, starch fractions, fermentation, glycemic index, bread, texture

## Abstract

This study aimed to evaluate the influence of sourdough fermentation on the estimated glycemic index (eGI), in vitro starch digestibility, and textural and sensory properties of eight experimentally prepared sourdough breads. Wheat and whole wheat flour bread samples were produced under different fermentation conditions (25 °C and 30 °C) and fermentation methods (type-1 and type-2). In type-1 fermentation, sourdough was obtained via spontaneous fermentation. Indigenous strains (*Lactobacillus brevis ELB99*, *Lactiplantibacillus plantarum ELB75*, and *Saccharomyces cerevisiae TGM55*) were used for type-2 fermentation. Fermentation type and temperature significantly affected eGI, the hydrolysis index (HI), the starch fraction, and the textural properties of the samples (*p* < 0.05). The resistant starch (RS) content increased after fermentation, while rapidly digestible starch (RDS), HI, and eGI decreased. RS values were significantly higher in type-2 than in type-1 at the same temperature for both flour types (*p* < 0.05). At 25 °C, RS values were higher in both fermentation types. In the white flour samples, eGI values were in the range of 60.8–78.94 and 62.10–78.94 for type-1 and type-2, respectively. The effect of fermentation type on eGI was insignificant (*p* < 0.05). In the whole flour samples, fermentation type and temperature significantly affected eGI (*p* < 0.05). The greatest eGI decreases were in whole wheat sourdough bread at 30 °C using type-2 (29.74%). The 30 °C and type-2 samples showed lower hardness and higher specific volume. This study suggests that fermentation type and temperature could affect the eGI and the textural and sensory properties of sourdough bread, and these factors should be considered during bread production. The findings also support the consumption of wheat and whole wheat breads produced by type-2 fermentation due to higher RS and slowly digestible starch (SDS) and lower RDS and eGI values.

## 1. Introduction

Bread is the most consumed food item in the world and the primary source of carbohydrates in European countries [1,2]. Bread is a grain-based food and part of a daily diet consisting of adequate carbohydrates, protein, dietary fiber, and vitamins. Bakery products, which are the main carbohydrate source of the daily diet, have high glycemic indexes, even if they are produced from whole grain [3].

After bread is consumed, the starch is often rapidly digested and absorbed, leading to hyperglycemia in individuals suffering from insulin resistance syndrome [4,5]. Including more rapidly digested carbohydrates in the daily diet may cause a rapid increase in the blood glucose level and a requirement for more insulin in the postprandial period. Furthermore, hyperglycemia is a risk factor that plays a role in the etiology of metabolic syndrome-related diseases [4].

Starch digestibility can be affected by many factors, including physiological factors, such as the binding of α-amylase to the substrate, gastric emptying, enzyme inhibitors, and the properties and viscosities of digestive enzymes [6]. Under optimal processing conditions, functional microorganisms can contribute to food functionality. Applying sourdough fermentation in bakery foods reduces glycemic responses in the end product and improves the dietary fiber complex [7].

In recent years, consumers have turned to various alternatives due to white bread’s high glycemic index and low fiber content. Sourdough bread has started to replace white bread due to its high resistant starch content, high mineral bioavailability, low glycemic index, and better sensory qualities [8]. In 2018, the sourdough market was estimated to be 2.4 billion USD.

Sourdough fermentation is the oldest method of dough fermentation and occurs with the help of yeast and lactic acid bacteria (LAB) [9]. It is particularly effective in low pH ranges (3.5–4.0) and reduces the glycemic index with soluble fiber [9]. Consumption of sourdough bread reduces postprandial blood glucose and insulin response. This mechanism could explain why organic acids, such as lactic and acetic acids, produced in sourdough lower its estimated glycemic index (eGI). Acetic acid appears to be associated with a delay in gastric emptying, whereas lactic acid induces interactions between starch and gluten during dough baking and reduces starch availability [7].

Many endogenous and exogenous elements affect the number of microorganisms (yeast and LAB), the presence of microbial species during the process, and the interaction between the species in sourdough [10]. Changes in technological factors, such as the amount of water and flour used for refreshment, fermentation time, and temperature, may directly affect the properties of sourdough [10].

Although previous studies have reported that sourdough fermentation helps reduce the glycemic index and can alter starch digestion [11,12], the effects of different fermentation conditions on bread properties have not been studied. In addition, more studies should be conducted on how indigenous starter cultures affect sourdough bread properties, especially eGI and resistant starch (RS).

To address this gap, the present study aimed to determine how different fermentation conditions—spontaneous fermentation vs. the addition of pure indigenous cultures (*Lactobacillus brevis ELB99*, *Lactiplantibacillus plantarum ELB75*, and *Saccharomyces cerevisiae TGM55*)—affect the glycemic index and starch digestibility. Thus, bread samples produced spontaneously or via inoculation with pure strains using different types of flour and at two different temperatures (25 °C and 30 °C) were analyzed. Dough properties, bread physical properties, chemical composition, in vitro starch digestion, and the estimated glycemic index were measured to provide healthier bread recommendations to the consumer.

## 2. Materials and Methods

### 2.1. Materials

Wheat flour, whole wheat flour, and drinking water were purchased from local producers in Turkey (Akınsoy Food Industry and Pınar Water and Beverage Industry, respectively). Invertase (from yeast, 300 U/mL, E-INVPD-2G), thermostable α-amylase (from *Bacillus licheniformis*, 3000 U/mL, E-BLAAM-10ML), amyloglucosidase (from *Aspergillus niger*, 3330 U/mL, E-AMGDF-10ML), and glucose oxidase-peroxidase (GOPOD) reagent were purchased from Megazyme (Wicklow, Ireland). Pepsin (from porcine gastric mucosa, 250 U/mL, P7000-100G), pancreatin (from porcine pancreas, 8 × USP specifications, P7545-100G), and guar gum were obtained from Sigma–Aldrich Co., LLC. (St. Louis, MO, USA).

### 2.2. Sourdough Fermentation

Fermentation conditions, including fermentation type, temperature, flour type, indigenous microorganism, and sample code, are given in Table 1. The bread samples were produced using wheat flour and whole wheat flour at two different fermentation temperatures (25 °C and 30 °C) and using two different sourdough fermentation methods (type-1 and type-2). In type-1 fermentation, sourdough was produced via spontaneous fermentation. In type-2 fermentation, indigenous strains (*Lactobacillus brevis ELB99*, *Lactiplantibacillus plantarum ELB75*, and *Saccharomyces cerevisiae TGM55*) were used.

For type-1 fermentation, 187.5 g of wheat flour and whole wheat flour and 112.5 mL of drinking water were mixed in a continuous high-speed mixer (60× *g*, dough mixing time of 5 min) to obtain 300 g of dough (dough yield (dough weight × 100/flour weight) 160) [13]. Sourdough was obtained by continuing the fermentation process via feeding three times every 24 h. After the first fermentation, sourdoughs were propagated (10%, *w*/*w*) into 168.75 g of flour and 101.25 mL of drinking water. Sourdough fermentation ended when the pH value reached 4.00 ± 0.20. The fermentation times for type-1 and type-2 were 96 h and 24 h, respectively.

For type-2 fermentation, *Lactobacillus brevis ELB99*, *Lactiplantibacillus plantarum ELB75,* and *Saccharomyces cerevisiae TGM55*, obtained from the Yıldız Technical University Food Engineering culture collection, were activated by pure cultures and mixed with 30 mL of culture mixture, 187.5 g wheat flour and whole wheat flour, and 82.5 mL of drinking water. For type-2 sourdough preparation, the same temperature conditions (25 °C and 30 °C) were applied for 24 h. Two replicates were prepared per each sourdough treatment. Each sample was coded based on the fermentation condition using numbers and letters.

The control wheat (CW) and whole wheat (CWW) bread samples were produced according to the following method: 100 g of wheat and whole wheat flour, 2 g of dry yeast, 1.5 g of salt, water (for 61.4 mL of wheat flour and for 64.1 mL of whole wheat flour) were mixed. After 30 min of mass fermentation, 160 g of bread was put into pans and kept in an air conditioning cabinet at 75% humidity and 30 °C for 45 min.

### 2.3. Dough Characterization

The microbiological properties of the sourdough were determined using a modified version of the method described by Bottani et al. [14]. At the beginning and end of the sourdough fermentation process, 10 g of dough and 90 mL of sterile peptone water were homogenized. MRS agar (de Man, Ragosa and Sharpe, Merck, Darmstadt, Germany) and SD agar (Sabouraud dextrose, Merck, Germany) were used for LAB and yeast enumeration. Dilution series were prepared according to the predicted concentration of the samples. The dilutions were inoculated, and then petri dishes were incubated in incubation cabinets for 48 h. The results were expressed as log CFU/g. A pH meter was used to measure the pH of the sample five times during the type-1 sourdough preparation (at 0 h, 24 h, 48 h, 72 h, and 96 h) and twice during the type-2 sourdough preparation (0 h and 24 h) (HANNA instrument HI2211, Darmstadt, Germany) [15].

### 2.4. Bread Production and Sampling

The flour was mixed with drinking water and salt (NaCl) as well as sourdough samples, which comprised 30% of the dough weight. The control samples were prepared without sourdough according to basic bread formulation practices. The sourdough bread was prepared using the same formulation but with slight modifications. All ingredients were kneaded in a spiral mixer (Arzum AR1066, Istanbul, Turkey) for 6 min at medium speed. The dough was divided into 160 g loaves and manually molded, and the loaves were proofed in pans. After resting for 30 min at room temperature, the dough was fermented at 30 °C and 75% relative humidity for 2 h in a proofing cabinet (Nuve TK252, Ankara, Turkey). Three loaves were prepared from each sourdough replicate. The dough was baked in an electric oven (Fimak, Konya, Turkey) for 45 min at a bottom temperature of 190 °C and a top temperature of 215 °C. Two replicates of bread were prepared per each sourdough treatment. The bread samples were cooled at room temperature for 2 h before further analysis. After physical analysis, the bread samples were ground using a laboratory mill (PX-MFC 90 D, Kinematica, Malters, *Switzerland*) and weighed into falcon test tubes. The samples, consisting of a homogeneous mix of crumb and crust, were stored at 4 °C until further analysis.

### 2.5. Bread Physical Properties

The specific volume (SV) of the bread samples was measured using the rapeseed displacement method [15]. The L*, a*, and b* values of the crust and crumb color of the bread samples were measured using a chromameter (CR-100 Konica Minolta, Osaka, Japan). L* indicates luminosity, while a* and b* indicate chromaticity on a green (−) to red (+) axis and a blue (−) to yellow (+) axis, respectively. The texture (hardness, springiness, cohesiveness, chewiness, and resilience) of uniform (25 mm-thick) slices of bread was determined using a texture analyzer (TA.XT2 Plus, Godalming, UK).

### 2.6. Chemical Composition of Bread Samples

The bread samples were analyzed to determine their levels of moisture (Association of Official Analytical Chemists (AOAC) International method No. 925.10), ash (AOAC International method 923.03), protein (Kjeldahl method, AOAC International method 920.87), fat content (Soxhlet extraction method, AOAC International method 945.16), and total dietary fiber (AOAC International method 985.29) [16]. Available carbohydrates (g/100 g) were calculated as the difference between the protein, fat, ash, moisture, and dietary fiber content in the bread. The energy value was calculated using the following equation [12]:

EnergyValue(kcal)=(protein×4)+(fat×9)+(carbohydrates×4)+(fibers×2)

The total starch of the bread samples was determined using a slightly modified version of the Goñi et al. [17] method. Briefly, 0.1 g of the sample and 0.2 mL of aqueous ethanol (80%, *v*/*v*) were vortexed to provide dispersion. Then, a 2 mL 2 M KOH solution was added and mixed with a magnetic stirrer on an iced water bath for 20 min. Then, 0.1 mL of thermostable α-amylase and 0.1 mL of amyloglucosidase were added to each sample, and the samples were incubated at 50 °C for 30 min. Next, the final volume was adjusted to 50 mL with deionized water, and the samples were centrifuged at 4000× *g* for 5 min. Finally, the glucose content of the supernatant was determined using an assay kit GOPOD-format K-GLUC (Megazyme International Ireland Ltd., Wicklow, Ireland) by a spectrophotometry (Shimadzu UV-1800, Kyoto, Japan) at a wavelength of 510 nm.

### 2.7. eGI and Starch Digestibility of Bread Samples

The eGI of the bread samples was determined using slightly modified versions of the methods of Englyst et al. [18] and Yaman et al. [19]. To determine the in vitro glycemic index, 1 g of homogenized bread sample was mixed with 5 mL of deionized water. Then, 10 mL of pepsin-guar gum solution was added to the sample and incubated at 37 °C for 30 min in a shaking water bath (175 strokes/min). After incubation, 0.5 M of sodium acetate solution (5.0 mL) was added, and the pH was adjusted to between 5 and 5.25. An enzyme solution, which included pancreatin and amyloglucosidase (13.4 U/mL), was added, and the volume was adjusted to 50 mL with deionized water. Then, the sample was incubated in a shaking water bath for 180 min. During incubation, 0.5 mL samples were taken at 20, 30, 60, 90, 120, and 180 min and placed in separate test tubes. The test tubes were placed in a boiling water bath for 5 min to enable the denaturation of the enzymes. Then, the final volume was adjusted to 5 mL using deionized water, and the samples were centrifuged at 4000× *g* for 5 min. After that, the glucose content of the supernatant was determined using an assay kit GOPOD-format K-GLUC (Megazyme International Ireland Ltd.) by a spectrophotometry (Shimadzu UV-1800, Japan) at a wavelength of 510 nm.

The eGI was calculated from the hydrolysis index (HI) value of each sample. The HI value was obtained by dividing the area under the hydrolysis curve of the white bread area obtained from the local market. The eGI was calculated using the formula below, as described by Goñi, Garcia-Alonso, and Saura-Calixto [17]:

GI = 39.71 + 0.549 × HI

Starch digestibility was determined using the same procedure. The glucose content of 0.5 mL samples taken at 20 and 120 min was measured and calculated according to the formula below:

$$\begin{array}{l} \mathrm{TS}(\mathrm{Total}\mathrm{starch})=\left(G_{\mathrm{TS}}\times F\times 0.9\times 100\right)/W\\ \mathrm{RDS}\left(\mathrm{Rapidly}\mathrm{digestible}\mathrm{stach}\right)=\left(G_{20}\times F\times 0.9\times 100\right)/W\\ \mathrm{SDS}\left(\mathrm{Slowly}\mathrm{digestible}\mathrm{strach}\right)=\left(\left(G_{120}-G_{20}\right)\times F\times 0.9\times 100\right)/W\\ \mathrm{RS}\left(\mathrm{Resistant}\mathrm{starch}\right)=\mathrm{TS}-\left(\mathrm{RDS}+\mathrm{SDS}\right)\\ G_{20}:\mathrm{Absorbance}\mathrm{value}\mathrm{for}\mathrm{glucose}\mathrm{after}20\min\mathrm{incubation}.\\ \begin{array}{l} G_{120}:\mathrm{Absorbance}\mathrm{value}\mathrm{for}\mathrm{glucose}\mathrm{after}120\min\mathrm{incubation}.\\ G_{\mathrm{TS}}:\mathrm{Absorbance}\mathrm{value}\mathrm{of}\mathrm{total}\mathrm{strach} \end{array}\\ F:100/\mathrm{GOPOD}\mathrm{absorbance}\\ W:\mathrm{Sample}\mathrm{weight}(\mathrm{mg}) \end{array}$$

An experimental factor of 0.9 was used to convert monosaccharides into polysaccharides. RS values of the bread samples were calculated using the difference between total starch glucose absorbance and glucose release at 120 min. TS and starch fraction were calculated as g/100 g wet samples.

### 2.8. Statistical Analysis

All analyses were performed at least in duplicate for each batch of sourdough (four analyses for each type of sourdough). The results were presented as mean and standard deviation (SD) values. Two-way analysis of variance (ANOVA) was conducted to evaluate any significant differences between means. Fermentation type and temperature were the studied parameters. IBM SPSS Statistics 24.0 (SPSS Inc., Chicago, IL, USA) was used to perform the statistical analyses. Significant differences (*p* < 0.05) were determined using Tukey’s multiple range test. All data are expressed as means of at least triplicate measurements.

## 3. Results and Discussion

### 3.1. Dough Properties and Characterization

Table 2 shows that fermentation type and temperature had significant effects (*p* < 0.05) on the microorganism count of the wheat and whole wheat sourdough. The microorganism count was higher in the type-2 fermentation at the same temperature values in both flour types. Similarly, in the same fermentation type, the fermentation condition at 30 °C showed a higher level of microorganism count than at 25 °C.

In the type-1 fermentation method, the LAB content of the dough ranged from <2 log CFU/g to 3.18 log CFU/g, and the LAB amount weighed between 9.08 and 9.57 log CFU/g. After feeding three times with flour and water and fermentation at the appropriate temperature, there was approximately a three-fold increase in the LAB counts of sample 1W30 with no change in the yeast count. As shown in Table 2, there was approximately a two-fold increase in the LAB content of sample 1WW30, and the yeast count was found to be <2 log CFU/g by suppressing the development of lactic acid bacteria. There was approximately a two-fold increase in the LAB content of sample 1W25, while the content of yeast did not change. In sample 1WW25, the LAB content was not initially detected but was 9.26 log CFU/g after fermentation.

In the type-2 fermentation method, the LAB content of the dough at 0 h ranged from 8.74 to 9.28 log CFU/g. This level reached 10.11 log CFU/g (2WW25) and 10.20 log CFU/g (2W25) in the dough obtained at 25 °C after fermentation, while the remaining samples reached 11.56 log CFU/g (2WW30) and 11.59 log CFU/g (2W30) at 30 °C after fermentation. The LAB counts obtained at 30 °C after fermentation were significantly higher than those obtained at 25 °C after fermentation (*p* < 0.05), while the initial LAB count was not significantly different for the two temperatures. As shown in Table 2, the yeast count at 0 h varied between 6.53 and 6.80 log CFU/g, and the yeast count increased on average by 1 log CFU/g regardless of the fermentation temperature. Overall, type-2 fermentation and 30 °C temperature conditions resulted in a higher microorganism count than type-1 fermentation and 25 °C temperature conditions.

Table 2 also shows the effects of fermentation type and temperature on the pH value of the sourdough samples. At 0 h, the dough prepared with whole wheat flour had a higher pH value than the dough prepared with wheat flour. The target pH value was reached within 96 h in type-1 fermentation and within 24 h in type-2 fermentation. In addition, the acidification rate was higher at 30 °C in both fermentation types than at 25 °C. Faster acidification as the temperature increased from 28 °C to 35 °C was also reported in [20]. Similar to the present findings, Bolarinwa et al. [21] reported that fermentation time and temperature were significantly affected by pH change and the acidification rate, which increased as the fermentation temperature increased. The fast dough acidification in type-2 fermentation at higher temperatures could be beneficial for industrial applications, such as inhibiting the growth of spontaneous, naturally occurring yeasts [22].

### 3.2. Bread Textural Properties

The textural properties—hardness, springiness, cohesiveness, chewiness, resilience, and specific volume—of the sourdough and control bread samples are presented in Table 3. For the wheat bread, type-1 and type-2 fermentation significantly increased the hardness and chewiness values and significantly decreased the springiness, cohesiveness, and resilience values (*p* < 0.05). Furthermore, the hardness and chewiness values of type-1 fermentation were significantly higher than those of type-2 fermentation (p < 0.05). In addition, the hardness and chewiness values were higher for the type-1 and type-2 fermentation samples at 25 °C than at 30 °C. There was a decrease in the springiness, cohesiveness, and resilience values compared to those in the control samples. While the springiness, cohesiveness, and resilience values were affected by the fermentation type, they were not significantly affected by the fermentation temperature (*p* > 0.05).

Similar to the wheat bread, type-1 and type-2 fermentation significantly increased the hardness and chewiness values and significantly decreased the springiness, cohesiveness, and resilience values of whole wheat bread. Type-1 fermentation and a temperature of 25 °C produced the highest hardness and chewiness values and the lowest springiness, cohesiveness, and resilience values compared to other fermentation conditions.

Increases in hardness and chewiness in sourdough bread were also reported by Alba et al. [23]. Similar to our study, Siepmann, Sousa de Almeida, Waszczynskyj, and Spier [20] reported that bread hardness increased with the addition of sourdough and an increase in fermentation temperature. Yildirim and Arici [8] reported that increasing the temperature from 25 °C to 35 °C significantly increased the hardness value. The resilience values of the wheat bread samples are similar to the values reported by Coda et al. [22].

Gluten protein plays a very important role in determining the texture of sourdough bread. Lactic acid produced by LAB can cause hydrolysis, swelling, and increased solubility in gluten protein. The exopolysaccharides produced by some LAB strains are another important factor affecting bread texture. This difference in bread texture during fermentation may be due to physicochemical changes in the gluten protein as a result of increased acidity [24]. Meanwhile, the type-2 sourdough bread had a higher specific volume (*p* < 0.05) than the type-1 sourdough bread. The pure cultures added to the formulation in type-2 provided a greater volume increase in the bread samples compared to spontaneous fermentation in type-1. For each flour type, a significant increase was observed in the specific volume value with increased temperature (*p* < 0.05). A similar result was reported by Yildirim and Arici [8].

**Figure 1:**
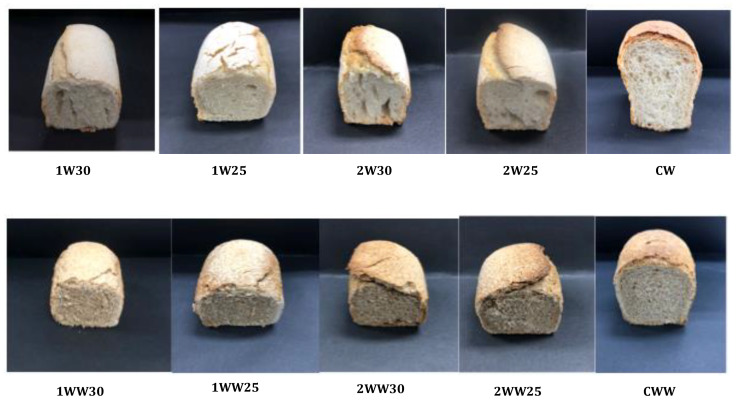
Visual appearance of sourdough and control breads.

The crust and crumb colors of the bread samples are presented in Table 4, and the visual appearance of the samples is shown in Figure 1. For the wheat bread, fermentation type and temperature significantly affected the crust color (*p* < 0.05). Type-1 fermentation and a 30 °C temperature resulted in a higher L value than type-2 fermentation and a 25 °C temperature. Temperature had an insignificant effect on the a* value in type-2 fermentation, while, in type-1 fermentation, the a* value obtained at 25 °C was significantly higher than that obtained at 30 °C. Meanwhile, a higher b* value was obtained at 25 °C in type-1 fermentation and at 30 °C in type-2 fermentation. Fermentation temperature had an insignificant effect on crumb color in the wheat bread.

Regarding the crust color of whole wheat bread, the highest L* value was obtained from type-1 fermentation and a 30 °C temperature. Temperature and fermentation type significantly affected the a* value (*p* < 0.05), and the highest a* value was obtained in type-2 fermentation at 30 °C. The highest value of b* was obtained in type-1 fermentation at 30 °C. Fermentation temperature had an insignificant effect on crumb color in the whole wheat bread.

### 3.3. Chemical Composition of Bread Samples

The chemical composition of the bread samples is presented in Table 5. Although the effects of fermentation type and temperature on the nutritional composition of the bread samples were significant (*p* < 0.05), a clear trend was not observed. In type-1 fermentation, dietary fiber increased in the wheat and whole wheat bread samples as the fermentation temperature increased, while the reverse trend was observed in type-2 fermentation. An increase in dietary fiber with sourdough fermentation was reported in [25,26]. The energy values of the bread samples showed a negative trend with dietary fiber content. The sample with low dietary fiber showed a higher energy value. Both fermentation type and temperature significantly affected energy value (*p* < 0.05). However, there was no clear trend in fermentation condition and energy value.

### 3.4. Starch Fractions

The RS, rapidly digestible starch (RDS), and slowly digestible starch (SDS) values of the sourdough and control bread samples are presented in Table 6. For all fermentation conditions and both types of flour, RS was significantly higher in the sourdough bread samples than in the control samples (*p* < 0.05). Organic acids, especially lactic acid, which is formed as a result of sourdough fermentation, cause an increase in the RS ratio [27,28]. The effects of fermentation type and temperature on RS were significant (*p* < 0.05). RS values obtained from type-2 fermentation were significantly higher than those obtained from type-1 fermentation. At 25 °C, RS values were higher in both fermentation types. Higher RS values at low fermentation temperatures were also reported by Liljeberg et al. [29].

Since SDS and RDS have potential health effects linked to glucose metabolism, diabetes management, and satiety improvement [30], their values should be considered in fermentation condition selection and starter culture determination. Especially in type-1 fermentation, lower RDS and higher SDS values were obtained at 30 °C. As shown in Table 6, RDS was highest in the control bread sample (CW) with a value of 23.35 g/100 g starch.

Because the rapid digestion of starch causes an increased glycemic response, products with low RDS may be preferred by diabetic patients [4]. RDS was lowest in the 2WW30 and 2WW25 samples (8.32 and 8.24 g/100 g starch, respectively), indicating that type-2 fermentation led to a decrease in starch digestibility. Adding sourdough seems to be an effective strategy for reducing RDS. Compared to the CWW and 2WW25 samples, which were prepared with whole wheat flour, the addition of sourdough reduces the amount of RDS by approximately half. As seen in Table 6, SDS was highest in the 1WW30 sample (22.74 g/100 g starch), followed by the 1W30 sample (21.31 g/100 g starch).

### 3.5. Estimated eGI and HI of Bread Samples

**Figure 2:**
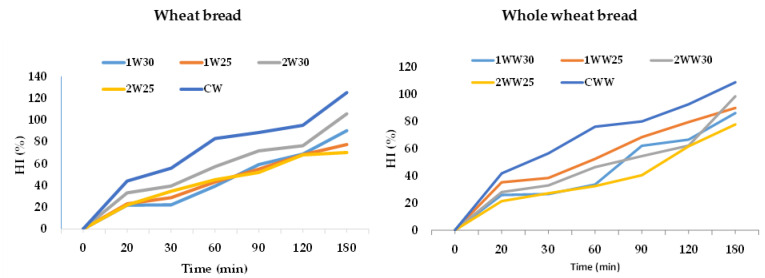
The hydrolysis curves for the sourdough and control bread samples.

The eGI and HI values of the bread samples are shown in Table 6. HI ranged from 68.29 to 133.08. The lowest HI values belonged to the 2WW30 sample, similar to the eGI values. The hydrolysis curves for the sourdough and control bread samples are shown in Figure 2. As evident in Table 6, fermentation type and fermentation temperature both significantly affected eGI and HI. Under all fermentation conditions, the eGI and HI values were significantly lower in both flour types compared to the control samples (*p* < 0.05). In the whole wheat bread samples, the decreases in eGI and HI in type-2 fermentation at 30 °C were significantly larger than those in type-1 fermentation at 25 °C. This result can be explained by the higher level of lactic acid produced under these fermentation conditions. The control bread samples had statistically higher eGI values than the sourdough bread samples (*p* < 0.05). Specifically, the CW and CWW samples had values of 78.94 and 76.93, respectively. The efficiency of adding sourdough was shown by comparing the controls of the same flour type that were prepared without sourdough. The addition of sourdough yielded a eGI value of 54.05 in the 2W30 sample, which was a decrease of 29.74%, and there was a decrease of 25.61% in the 2WW25 sample.

When comparing the type-1 and type-2 fermentation methods, the addition of pure culture decreased the eGI values more effectively than spontaneous fermentation, especially in the whole wheat bread. The decrease in the eGI values in the wheat bread samples obtained by applying the type-1 and type-2 fermentation methods was less than in the whole wheat bread samples. As shown in the literature, in vitro starch digestion and in vivo blood glucose levels in healthy individuals both decreased significantly after consumption of sourdough bread compared with control bread [28]. The 2WW30 and 2WW25 samples, which had the greatest eGI decreases among all bread samples, also had the highest RS content (16.93 and 15.22 g/100 g, respectively).

When evaluating the eGI values of the type-1 sourdough bread samples, it was clear that the values were not only related to RS but also changed according to the content of the RDS and SDS fractions. Besides the effects of different fractions of starch on eGI, the main role in the reduction of the glycemic index may be due to the presence of organic acids [20]. Physiological mechanisms change the acute effects of acids while lactic acid lowers the starch digestion rate, and acetic and propionic acids lower the small intestine pH, which may reduce the activity of starch digestion enzymes such as α-amylase and α-glucosides [31]. The fact that the microbiological diversity in the spontaneous (type-1) sourdough samples was different than in the type-2 fermentation samples may have caused a difference in the characteristics of sourdough obtained and in the glycemic response of the final product.

## 4. Conclusions

Sourdough was found to be a helpful agent for regulating the digestibility of starch and, consequently, reducing eGI in bread products. The eGI of sourdough bread was statistically lower than that of the control bread (*p* < 0.05). The greatest decreases in eGI were in the whole wheat sourdough bread samples obtained at 30 °C using the type-2 fermentation method. Furthermore, when the type-2 and type-1 sourdough fermentation methods were compared, the type-2 sourdough samples with whole wheat flour had the most effective increase in RS content.

Sourdough bread can be recommended as a part of a daily diet due to its low glycemic response. Studies on the health effects of sourdough are expected to continue in order to investigate different fermentation conditions (temperature-duration combinations) and in vivo and in vitro correlations.

## Figures and Tables

**Table 1:** Type-1 and type-2 fermentation conditions and sample code.

Fermentation Type	Fermentation Temperature	Material	Sample Code
Type-1 (spontaneous)	25 °C	Wheat flour	1W25
		Whole wheat flour	1WW25
	30 °C	Wheat flour	1W30
		Whole wheat flour	1WW30
Type-2 (addition mix of *Lactobacillus brevis ELB99*, *Lactiplantibacillus plantarum ELB75*, *Saccharomyces cerevisiae*)	25 °C	Wheat flour	2W25
		Whole wheat flour	2WW25
	30 °C	Wheat flour	2W30
		Whole wheat flour	2WW30
Not fermented		Control wheat flour	CW
Not fermented		Control whole wheat flour	CWW

**Table 2:** The effect of fermentation types and temperature on microbiological properties of sourdough.

		LAB (log CFU/g)		Yeast		pH			LAB		Yeast		pH	
	Sample	0th h	96th h	0 th	96th h	0th h	96th h	Sample	0th h	96th h	0th h	96th h	0th h	96th h
	Wheat flour samples							Whole wheat flour samples						
Type-1	1W30	2.60 ± 0.14 ^B,a^	9.57 ± 0.05 ^B,a^	<2 ^B,a^	<2 ^Ba^	6.19 ± 0.0 ^A,b^	3.87 ± 0.02 ^A,b^	1WW30	3.18 ± 0.08 ^B,a^	9.36 ± 0.12 ^B,a^	3.23 ± 0.06 ^B,a^	<2 ^B,a^	6.32 ± 0.03 ^A,b^	3.98 ± 0.00 ^A,b^
	1W25	3.04 ± 0.05 ^B,a^	9.08 ± 0.11 ^B,b^	<2 ^B,a^	<2 ^Ba^	6.29 ± 0.01 ^A,a^	3.98 ± 0.00 ^A,a^	1WW25	< 2 ^B,b^	9.26 ± 0.09 ^B,a^	3.08 ± 0.08 ^B,a^	<2 ^B,a^	6.47 ± 0.02 ^A,a^	4.12 ± 0.03 ^A,a^
	**Sample**	**0th h**	**24th h**	**0th h**	**24th h**	**0th h**	**24th h**	**Sample**	**0th h**	**24th h**	**0th h**	**24th h**	**0th h**	**24th h**
Type-2	2W30	9.28 ± 0.23 ^A,a^	11.59 ± 0.11 ^A,a^	6.80 ± 0.12 ^A,a^	7.99 ± 0.04 ^A,a^	5.91 ± 0.00 ^B,a^	3.83 ± 0.01 ^A,b^	2WW30	9.08 ± 0.12 ^A,a^	11.56 ± 0.04 ^A,a^	6.72 ± 0.02 ^A,a^	7.59 ± 0.04 ^A,a^	6.22 ± 0.00 ^B,b^	4.00 ± 0.01 ^A,b^
	2W25	9.18 ± 0.03 ^A,a^	10.20 ± 0.27 ^A,b^	6.76 ± 0.15 ^A,a^	7.38 ± 0.06 ^A,b^	5.93 ± 0.02 ^B,a^	3.97 ± 0.03 ^A,a^	2WW25	8.74 ± 0.07 ^A,b^	10.11 ± 0.19 ^A,b^	6.53 ± 0.08 ^A,a^	7.30 ± 0.21 ^A,a^	6.32 ± 0.00 ^B,a^	4.18 ± 0.01 ^A,a^

The mean value ± standard deviations of quadruplet analysis are given. LAB: lactic acid bacteria, CFU: colony-forming unit. A–B: Different uppercase letter in same column indicates effect of fermentation type at same temperature, a–b: different lowercase letter in same column indicates effect of temperature for same fermentation types (*p* < 0.05).

**Table 3:** Physical properties of sourdough and control bread samples.

Sample	Hardness (N)	Springiness	Cohesiveness	Chewiness (N)	Resilience	Specific Volume (cm^3^/g)
Wheat flour breads						
1W30	27.98 ± 0.74 ^A,b^	0.92 ± 0.03 ^A,b^	0.76 ± 0.00 ^B,b^	19.60 ± 0.46 ^A,b^	0.42 ± 0.01 ^B,b^	1.53 ± 0.01 ^B,b^
1W25	47.24 ± 0.29 ^A,a^	0.89 ± 0.00 ^B,b^	0.76 ± 0.02 ^B,b^	28.00 ± 0.58 ^A,a^	0.45 ± 0.01 ^B,a,b^	1.38 ± 0.01 ^B,c^
CW	5.37 ± 0.44 ^c^	1.30 ± 0.09 ^a^	0.84 ± 0.00 ^a^	5.89 ± 0.13 ^c^	0.49 ± 0.00 ^a^	3.55 ± 0.03 ^a^
2W30	10.71 ± 0.22 ^B,b^	0.97 ± 0.01 ^A,b^	0.87 ± 0.01 ^A,a^	9.02 ± 0.14 ^B,b^	0.52 ± 0.03 ^A,a^	2.16 ± 0.01 ^A,b^
2W25	12.74 ± 0.51 ^B,a^	1.13 ± 0.02 ^A,a,b^	0.87 ± 0.00 ^A,a^	12.30 ± 0.03 ^B,a^	0.55 ± 0.00 ^A,a^	2.02 ± 0.00 ^A,c^
CW	5.37 ± 0.44 ^c^	1.30 ± 0.09 ^a^	0.84 ± 0.00 ^a^	5.89 ± 0.13 ^c^	0.49 ± 0.00 ^a,b^	3.55 ± 0.03 ^a^
Whole wheat flour breads						
1WW30	45.78 ± 0.01 ^A,b^	0.75 ± 0.00 ^A,b^	0.60 ± 0.01 ^B,b^	20.29 ± 1.00 ^A,a^	0.26 ± 0.02 ^B,b^	1.41 ± 0.02 ^B,b^
1WW25	54.25 ± 0.56 ^A,a^	0.75 ± 0.01 ^B,b^	0.54 ± 0.02 ^B,c^	21.87 ± 0.66 ^A,a^	0.24 ± 0.00 ^B,b^	1.27 ± 0.00 ^B,c^
CWW	19.02 ± 1.70 ^c^	0.92 ± 0.01 ^a,b^	0.73 ± 0.01 ^a^	12.83 ± 1.25 ^b^	0.36 ± 0.01 ^a^	2.54 ± 0.12 ^a^
2WW30	25.35 ± 1.60 ^B,b^	0.87 ± 0.02 ^A,a,b^	0.74 ± 0.01 ^A,a^	16.23 ± 0.5 ^B,b^	0.38 ± 0.01 ^A,a^	2.04 ± 0.03 ^A,b^
2WW25	33.63 ± 0.79 ^B,a^	0.87 ± 0.01 ^A,a^	0.73 ± 0.00 ^A,a^	21.21 ± 0.59 ^A,a^	0.38 ± 0.00 ^A,a^	1.80 ± 0.02 ^A,c^
CWW	19.02 ± 1.70 ^c^	0.92 ± 0.01 ^a,b^	0.73 ± 0.01 ^a^	12.83 ± 1.25 ^d,c^	0.36 ± 0.01 ^a^	2.54 ± 0.12 ^a^

The mean value ± standard deviations of quadruplet analysis are given. A–B: Different uppercase letter in same column indicates effect of fermentation type at same temperature, a–c: different lowercase letter in same column indicates effect of temperature for same fermentation types (*p* < 0.05).

**Table 4:** Color properties of the breads.

Sample	Crust Colour			Crumb Colour		
	L*	a*	b*	L*	a*	b*
Wheat flour breads						
1W30	75.21 ± 0.09 ^A,a^	−0.81 ± 0.57 ^B,b^	25.02 ± 1.13 ^B,c^	66.76 ± 0.42 ^A,a^	−2.920.19 ^A,b^	19.68 ± 0.47 ^A,a^
1W25	61.87 ± 0.54 ^A,b^	6.81 ± 0.65 ^B,a^	34.96 ± 0.39 ^A,a^	65.2 ± 0.34 ^Aa^	−3.23 ± 0.12 ^A,b^	19.04 ± 0.43 ^A,a^
CW	61.93 ± 0.98 ^b^	5.64 ± 0.13 ^a^	30.82 ± 0.31 ^b^	45.90 ± 1.12 ^b^	9.72 ± 1.36 ^a^	20.71 ± 2.25 ^a^
W30	61.70 ± 1.20 ^B,a^	10.26 ± 0.99 ^A,a^	29.53 ± 0.83 ^A,a^	67.98 ± 0.44 ^A,a^	−3.39 ± 0.05 ^A,b^	19.38 ± 0.00 ^A,a^
2W25	57.10 ± 0.80 ^A,b^	10.33 ± 1.01 ^A,a^	28.81 ± 3.39 ^B,a,b^	65.81 ± 0.36 ^A,a^	−3.44 ± 0.03 ^A,b^	17.40 ± 0.51 ^A,a^
CW	61.93 ± 0.98 ^a^	5.64 ± 0.13 ^b^	30.82 ± 0.31 ^a^	45.90 ± 1.12 ^b^	9.72 ± 1.36 ^a^	20.71 ± 2.25 ^a^
Whole wheat flour breads						
1WW30	62.34 ± 1.38 ^A,a^	3.82 ± 0.39 ^B,c^	26.2 ± 0.23 ^A,b^	51.12 ± 0.40 ^B,a^	3.85 ± 0.12 ^A,b^	20.40 ± 0.02 ^A,b^
1WW25	41.98 ± 0.45 ^B,c^	10.46 ± 0.51 ^A,a^	20.12 ± 0.47 ^B,b^	51.9 ± 0.49 ^A,a^	3.46 ± 0.28 ^A,b^	20.32 ± 0.29 ^A,b^
CWW	57.16 ± 0.73 ^b^	7.43 ± 0.06 ^a,b^	27.83 ± 0.47 ^a^	50.23 ± 0.36 ^a^	9.57 ± 0.04 ^a^	26.39 ± 0.96 ^a^
2WW30	48.33 ± 0.05 ^A,b^	9.87 ± 0.55 ^A,b,c^	24.45 ± 0.12 ^A,b^	56.09 ± 0.18 ^A,a^	3.47 ± 0.10 ^A,b^	21.53 ± 0.20 ^A,b^
2WW25	47.96 ± 0.29 ^A,b^	11.03 ± 0.55 ^A,a^	23.15 ± 0.56 ^A,b^	57.86 ± 0.33 ^A,a^	2.77 ± 0.15 ^A,b^	20.39 ± 0.04 ^A,b^
CWW	57.16 ± 0.73 ^a^	7.43 ± 0.06 ^c^	27.83 ± 0.47 ^a^	50.23 ± 0.36 ^b^	9.57 ± 0.04 ^a^	26.39 ± 0.96 ^a^

The mean value ± standard deviations of quadruplet analysis are given. A–B: Different uppercase letter in same column indicates effect of fermentation type at same temperature, a–c: different lowercase letter in same column indicates effect of temperature for same fermentation types (*p* < 0.05).

**Table 5:** Chemical composition of samples.

Samples	Moisture (g/100 g)	Ash (g/100 g)	Protein (g/100 g)	Fat (g/100 g)	Dietary Fiber (g/100 g)	Energy (kcal)
Wheat flour breads						
1W30	30.4 ± 0.36 ^A,a^	0.84 ± 0.03 ^B,b^	8.9 ± 0.03 ^B,c^	1.48 ± 0.01 ^A,b^	12.43 ± 0.15 ^A,a^	257.59 ± 1.78 ^B,b,c^
1W25	31.2 ± 1.06 ^A,a^	1.39 ± 0.04 ^A,a^	9.84 ± 0.01 ^A,a^	1.23 ± 0.00 ^B,c^	11.47 ± 0.17 ^A,b^	252.89 ± 4.07 ^B,c^
CW	30.08 ± 0.25 ^a^	1.33 ± 0.00 d^a^	9.15 ± 0.02 ^b^	1.88 ± 0.03 ^a^	9.23 ± 0.18 ^c^	265.30 ± 0.51 ^a^
2W30	26.61 ± 0.18 ^B,b^	1.43 ± 0.01 ^A,b^	9.03 ± 0.03 ^A,b^	1.35 ± 0.01 ^B,c^	6.48 ± 0.25 ^B,b^	281.65 ± 0.27 ^A,a^
2W25	30.04 ± 0.06 ^A,a^	1.46 ± 0.04 ^A,b^	8.37 ± 0.03 ^B,c^	1.69 ± 0.06 ^A,b^	9.3 ± 0.25 ^B,a^	263.85 ± 0.74 ^A,b^
CW	30.08 ± 0.25 ^a^	1.33 ± 0.00 ^a^	9.15 ± 0.02 ^a^	1.88 ± 0.03 ^a^	9.23 ± 0.18 ^a^	265.30 ± 0.51 ^b^
Whole wheat flour breads						
1WW30	30.8 ± 0.14 ^A,c^	1.78 ± 0.08 ^A,a^	8.99 ± 0.01 ^A,c^	1.72 ± 0.01 ^A,c^	17.76 ± 0.28 ^A,a^	242.74 ± 0.87 ^B,b^
1WW25	32.81 ± 0.08 ^B,b^	1.64 ± 0.04 ^B,b^	9.27 ± 0.07 ^A,b^	1.87 ± 0.02 ^A,b,c^	12.37 ± 0.10 ^A,c^	246.79 ± 0.26 ^B,a^
CWW	34.7 ± 0.25 ^a^	1.28 ± 0.01 ^c^	9.46 ± 0.02 ^a^	2.02 ± 0.01 ^a^	16.65 ± 0.42 ^b^	232.90 ± 1.77 ^b^
2WW30	28.24 ± 0.09 ^B,c^	1.97 ± 0.02 ^A,a^	8.68 ± 0.01 ^B,c^	1.76 ± 0.04 ^A,c^	11.95 ± 0.03 ^B,b^	264.10 ± 0.61 ^A,a^
2WW25	31.23 ± 0.18 ^B,c^	1.90 ± 0.01 ^A,a^	8.89 ± 0.01 ^A,b^	1.77 ± 0.01 ^B,b^	8.87 ± 0.17 ^B,c^	258.53 ± 0.98 ^A,b^
CWW	34.7 ± 0.25 ^a^	1.28 ± 0.01 ^b^	9.46 ± 0.02 ^a^	2.02 ± 0.01 ^a^	16.65 ± 0.42 ^a^	232.90 ± 1.77 ^c^

The mean value ± standard deviations of quadruplet analysis are given. A–B: Different uppercase letter in same column indicates effect of fermentation type at same temperature, a–c: different lowercase letter in same column indicates effect of temperature for same fermentation types (*p* < 0.05).

**Table 6:** Starch fractions of bread samples.

Samples	RS (g/100 g)	RDS (g/100 g)	SDS (g/100 g)	TS (g/100 g)	HI	eGI
	Wheat flour breads					
1W30	8.88 ± 0.52 ^B,b^	11.56 ± 0.62 ^B,c^	24.67 ± 0.94 ^A,a^	45.35 ± 0.13 ^A,b^	91.08 ± 2.84 ^A,c^	60.8 ± 1.09 ^A,c^
1W25	10.61 ± 0.75 ^B,a^	15.39 ± 0.58 ^A,b^	21.31 ± 0.06 ^A,b^	47.36 ± 0.84 ^A,a,b^	93.97 ± 0.48 ^A,b,c^	63.91 ± 0.18 ^A,b^
CW	2.35 ± 0.95 ^c^	23.35 ± 0.91 ^a^	23.95 ± 1.20 ^b^	49.99 ± 1.17 ^a^	133.08 ± 2.60 ^a^	78.94 ± 0.99 ^a^
2W30	11.77 ± 0.38 ^A,b^	12.85 ± 0.74 ^A,b^	22.26 ± 0.03 ^B,a^	47.06 ± 1.99 ^A,a^	92.95 ± 2.16 ^A,b^	63.52 ± 0.83 ^A,b^
2W25	13.66 ± 0.33 ^A,a^	13.12 ± 0.06 ^B,b^	22.69 ± 0.75 ^A,a^	49.69 ± 0.25 ^A,a^	89.26 ± 0.62 ^B,b^	62.1 ± 0.24 ^A,b^
CW	2.35 ± 0.95 ^c^	23.35 ± 0.91 ^a^	23.95 ± 1.20 ^b^	49.99 ± 1.17 ^a^	133.08 ± 2.60 ^a^	78.94 ± 0.99 ^a^
	Whole wheat flour breads					
1WW30	5.12 ± 0.22 ^B,b^	9.53 ± 0.90 ^A,b^	22.74 ± 0.54 ^A,a^	37.66 ± 1.57 ^A,a^	87.49 ± 2.06 ^A,c^	61.42 ± 0.79 ^A,c^
1WW25	6.07 ± 0.96 ^B,a^	14.97 ± 0.24 ^A,a^	17.11 ± 0.18 ^A,c^	38.19 ± 1.33 ^A,a^	100.45 ± 1.99 ^A,b^	66.39 ± 0.76 ^A,b^
CWW	2.86 ± 0.65 ^c^	16.14 ± 0.92 ^a^	20.58 ± 1.37 ^b^	39.66 ± 0.48 ^a^	127.85 ± 0.64 ^a^	76.93 ± 0.25 ^a^
2WW30	16.93 ± 0.71 ^A,a^	8.32 ± 1.10 ^B,b^	11.77 ± 0.93 ^A,c^	36.66 ± 1.57 ^A,a^	68.29 ± 0.8 ^B,c^	54.05 ± 0.31 ^B,c^
2WW25	15.22 ± 1.25 ^A,b^	8.24 ± 0.80 ^B,b^	15.30 ± 0.64 ^A,b^	39.03 ± 1.24 ^A,a^	76.59 ± 0.94 ^B,b^	57.23 ± 0.36 ^B,b^
CWW	2.86 ± 0.65 ^c^	16.14 ± 0.92 ^a^	20.58 ± 1.37 ^b^	39.66 ± 0.48 ^a^	127.85 ± 0.64 ^a^	76.93 ± 0.25 ^a^

**RS**: resistant starch, **RDS:** rapidly digestible starch, **SDS:** slowly digestible starch, **HI**: hydrolysis index, **eGI**: estimated glycemic index. The mean value ± standard deviations of quadruplet analysis are given. A–B: Different uppercase letter in same column indicates effect of fermentation type at same temperature, a–c: different lowercase letter in same column indicates effect of temperature for same fermentation types (*p* < 0.05).

## Data Availability

The data presented in this study are available on request from the corresponding author.
